# Craniocerebral metastases from a supraglottic squamous cell carcinoma: A case report and literature review

**DOI:** 10.1002/ccr3.2480

**Published:** 2019-10-02

**Authors:** Moisés León‐Ruiz, Julián Benito‐León

**Affiliations:** ^1^ Department of Neurology Hospital Universitario Príncipe de Asturias Alcalá de Henares, Madrid Spain; ^2^ Department of Neurology Hospital Universitario 12 de Octubre Madrid Spain; ^3^ Department of Medicine Faculty of Medicine Universidad Complutense de Madrid Madrid Spain; ^4^ Centro de Investigación Biomédica en Red sobre Enfermedades Neurodegenerativas (CIBERNED) Madrid Spain

**Keywords:** craniocerebral, metastases, seizure, squamous cell carcinoma, supraglottic

## Abstract

Craniocerebral metastases as the initial spread of supraglottic squamous cell carcinoma (SCC) are exceptional. The presence of several months' history of dysphagia, dyspnea, cachexia, tobacco/alcohol abuse, and seizure(s) is suspicious of craniocerebral metastases from an advanced‐stage supraglottic SCC. Physicians should be aware since early diagnosis and treatment may increase patient survival.

## Graphical abstract

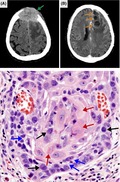

## 1 INTRODUCTION

Squamous cell carcinoma (SCC) represents more than 90% of tumours arising from the larynx. Tobacco and alcohol consumption are described as the main risk factors for laryngeal SCC development. Locoregional spreading through contiguous structures and lymphatic extension is the most frequent dissemination pattern for laryngeal SCC.^1^ Distant spreading, such as the brain, is rare and is generally supported by hematogenous dissemination, particularly in human papillomavirus‐related cases, or perineural invasion.^1^, ^2^

The incidence of symptomatic distant metastases from laryngeal SCC ranges from 1 to 4%, and the lung is among the most common sites of distant spread, followed by bone and liver.^1^ Intracranial metastases from laryngeal SCC are very unusual, which occur in less than 1% of all cases.^1^, ^2^

Of note, after conducting a literature review, we only found nine reported cases with intra‐ and extracranial metastases resulting from the spread of a supraglottic SCC.^1^, ^3^, ^4^, ^5^, ^6^, ^7^ We added our case and reviewed the most important data available from these reports on Table 1.

**Table 1:** Summary of clinical and outcome data of reported cases with intra‐ and extracranial metastases from supraglottic squamous cell carcinoma as the only primary tumour

Author and year of publication (in chronological order)	Number of patients	Sex	Age at diagnosis of metastases (y)	Treatment of primary tumour	Time to diagnose metastases (mo)	Symptoms	Location of metastases	Diagnosis	Surgery	Other therapies
Ahmad et al, 1984^3^	1	Male	70	RT	3	Diplopia Cheek numbness Amaurosis	Cavernous sinus	Clinical and radiological	No	RT
Warwick‐Brown and Cheesman, 1987^4^	1	Female	62	Total laryngectomy + pharyngectomy +RT	5	Personality change Retro‐orbital pain Facial numbness Proptosis Ptosis	Multifocal (cavernous sinus + brain)	Histopathological (postmortem)	No	No
Weiss et al, 1994^5^	1	Female	64	Total laryngectomy + partial pharyngectomy + bilateral neck dissection + RT	9	Diplopia VI CN palsy	Multifocal (pituitary gland + cavernous sinus)	Histopathological	Yes	RT
de Bree et al, 2001^6^	4	Female	53	Total laryngectomy + bilateral neck dissection	7	Diplopia Ptosis	Cavernous sinus	Histopathological	Yes	CT
		Male	75	NR	18	NR	Multifocal (brain + bone)	Clinical and radiological	No	RT
		Female	52	NR	8	NR	Brain	Clinical and radiological	No	RT
		Male	50	NR	NR	NR	Brain	Clinical and radiological	No	RT
Uzal et al, 2001^7^	1	Male	55	Partial laryngectomy + left neck dissection	9	Headache Diplopia Blurred vision Polydipsia Polyuria	Multifocal (pituitary gland + lung)	Histopathological	Yes	RT
Montano et al, 2018^1^, ^a^	1	Male	65	Total laryngectomy + bilateral neck dissection	36	Right hemiparesis	Multifocal (brain + lung)	Histopathological	Yes	RT + CT
Present case	1	Male	71	Total laryngectomy + bilateral neck dissection	6	Seizure	Multifocal (brain + skull)	Clinical, radiological, and histopathological	Yes	RT + CT

Note

Adapted from Montano et al, 2018.^1^

Abbreviations: CN, cranial nerve; CT, chemotherapy; NR, not reported; RT, radiotherapy.

a Follow‐up still ongoing at the time of the publication.

## 2 CASE REPORT

A 71‐year‐old man with a 50‐year history of tobacco and alcohol abuse presented to the emergency department with a focal to bilateral tonic‐clonic seizure, lasting 2‐3 minutes, followed by drowsiness and confusion. He had a 6‐month history of dysphagia, dyspnea, and 22‐kg weight loss, along with hoarseness since last month. His past medical history was otherwise normal.

On examination, he was cachectic and had an evident inspiratory stridor and a painless forehead lump. Apart from that, chest X‐ray and laboratory results were normal, except for an elevation of creatine kinase (553 IU/L) and white blood cell count (11.5 × 10^3^/µL), probably due to the seizure.

**Figure 1:**
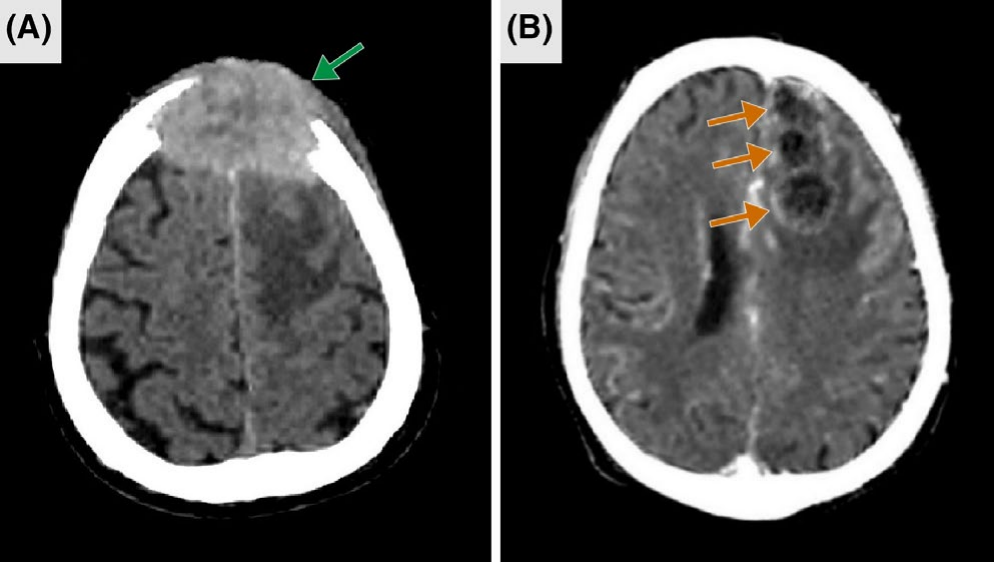
Brain computed tomography (CT). Axial CT scan without contrast reveals a frontal osteolytic lesion measuring 35 × 59 × 53 mm (green arrow) (A). Axial CT scan with contrast shows three ring‐enhancing lesions in the left frontoparietal area (orange arrows) (B)

Given a patient presenting with a first‐ever seizure, and considering the possible existence of an underlying brain neoplasm due to smoking, drinking, and weight‐loss patient's history, a brain computed tomography (CT) scan was obtained, showing a frontal osteolytic mass (Figure 1A) and ring‐enhancing lesions in the left frontoparietal region (Figure 1B).

## 3 OUTCOMES

We decided to commence the patient on levetiracetam administered intravenously. He became seizure‐free after starting at 1000 mg of levetiracetam twice daily.

**Figure 2:**
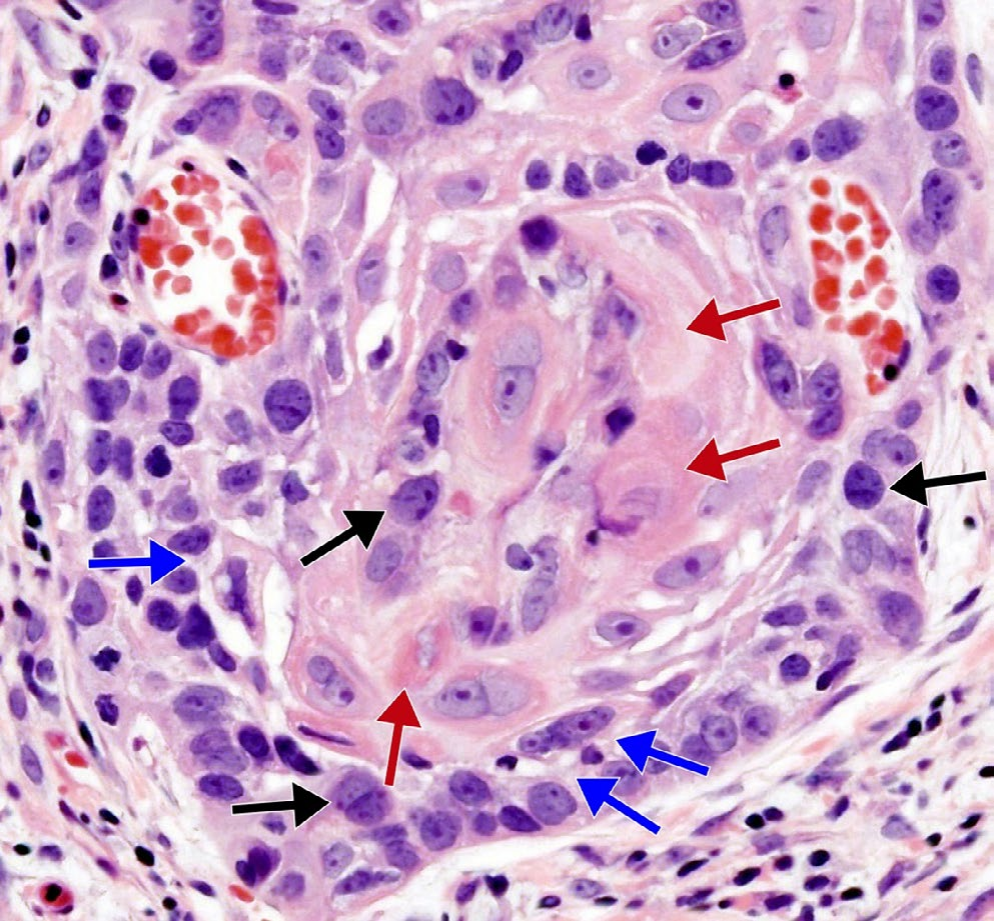
Histopathological image. Biopsy of the supraglottic lesion, one of the brain metastases, and the frontal bone mass discloses nests of atypical squamous cells with abnormal mitotic figures (black arrows), and intercellular bridges (blue arrows), surrounding central areas of cytoplasmic keratinization (red arrows) (hematoxylin and eosin staining, magnification 400×)

In light of patient's history, clinical evolution, and highlights from examination, diagnostic workup was completed with a laryngoscopy, which revealed a supraglottic exophytic lesion invading both the trachea and the esophagus. Pathological findings disclosed a poorly differentiated supraglottic SCC (Figure 2). Whole‐body CT imaging showed no evidence of further metastases at other locations.

He was diagnosed with supraglottic SCC (stage IVC) and underwent total laryngectomy, bilateral cervical lymphadenectomy, and adjuvant chemoradiotherapy. Unfortunately, he died on the fifth postoperative month due to acute respiratory failure from aspiration pneumonia.

## 4 DISCUSSION

There is a consensus that adults with an unprovoked first seizure should undergo neuroimaging to identify any process, which may be the seizure trigger. CT is usually easily available and rules out problems that deserve urgent attention in the emergency department and therefore preferred over magnetic resonance imaging (MRI).^8^, ^9^

Initiating antiepileptic drug (AED) therapy is recommended after the first seizure in patients at higher recurrence risk (eg, neurological deficit/s and/or brain lesion/s), because high‐risk patients with early AED treatment present a slightly more favorable long‐term outcome compared with those in whom it is delayed. Levetiracetam is indicated as first‐line monotherapy, being as efficacious as carbamazepine, for new‐onset focal seizures, with or without secondary generalization, but with no clinical hepatotoxicity, something to bear in mind before a patient with history of alcohol abuse.^10^

Computed tomography scanning and MRI are helpful studies for determining the spread of supraglottic cancer.^11^ Likewise, cranial CT, including both brain and bone window settings, constitutes a reliable and reproducible imaging technique for the evaluation and measurement of metastases in patients who are considered for treatment response assessment. The main advantages of CT are its availability, affordable cost, short scanning time, and the possibility of postscanning processing, whereas MRI is an expensive imaging modality for a serial follow‐up. The Response Evaluation Criteria in Solid Tumour (RECIST) guidelines were published in 2000. The RECIST criteria clarified the minimum size of measurable lesions, defined the number of lesions and applicable imaging techniques, and pinpointed the unidimensional measurements for the estimation of tumour burden.^12^

Furthermore, whole‐body multidetector computed tomography (WB‐MDCT) provides a high‐resolution imaging of cortical and trabecular bone with a short acquisition time, for approximately one minute. This technique provides a cost‐effective and patient‐friendly diagnostic tool, as well as an appropriate image quality to assess normal osseous architecture. The principal limitation of WB‐MDCT is its high radiation dose. Further studies regarding low‐radiation‐dose applications of this imaging modality are warranted.^13^

Moreover, cross‐sectional cranial imaging with CT and MRI could be useful for differentiating tumours from resembling lesions in renal osteodystrophy patients. In this scenario, we can find brown tumours presenting as focal reactive bone remodeling lesions with multiple cystic and/or mixed lesions, uremic leontiasis ossea characterized by serpiginous tunneling or channelling within the bone and poor visualization of the cortical bone, and dystrophic calcification with calcified soft‐tissue masses. It is recommended that clinical features be correlated with laboratory and radiological results to make a definitive diagnosis.^14^

In addition, MRI is essential for the differential diagnosis of a subglottic mass when suspecting laryngoscleroma, a chronic granulomatous inflammation caused by a Gram‐negative diplobacillus, *Klebsiella rhinoscleromatis,* specifically for evaluating the inferior extension of subglottic scleroma and its invasion into the trachea, along with distinguishing between sclerotic and granulomatous stages.^15^

On the other hand, it is worth mentioning a state‐of‐the‐art MRI technique such as arterial spin labeling (ASL), which does not require an exogenous contrast agent administration. Besides, it is noninvasive, nonionizing radiation, repeated safe, and able to supply quantitative values. It has a role in the characterization and grading of brain tumours, differentiation from mimicking lesions, pretreatment assessment, monitoring of treatment response, and discrimination of tumour recurrence from posttreatment changes. Its limitations include a low signal‐to‐noise ratio of tumour blood flow (TBF) maps and the potential for systematic measurement errors because of a prolonged transit delay between the tagging area and the imaging slice. Further research addressing parameters standardization of different ASL vendors is needed.^16^

The key clinical feature of this case is the conjunction of patient's history and neuroimaging and histopathological findings. Supraglottic cancer causes late‐onset symptoms, including dysphagia, trouble breathing, and voice changes, so is often diagnosed in evolved stages and may present with metastases in distant organs, owing to rich lymphatic supply in the supraglottic larynx.^11^, ^17^, ^18^

There is no standardized treatment for advanced (stages III and IV) supraglottic tumours,^1^, ^18^ but it can include total laryngectomy, bilateral neck dissections, radiotherapy, and/or chemotherapy, depending on each specific case.^11^, ^18^, ^19^, ^20^

Ultimately, brain metastases are a very rare initial spread due to laryngeal SCC. Although this occurrence has been adequately published in scientific literature,^1^, ^2^ to our knowledge, this is the first published case to show intra‐ and extracranial clinical metastases, in the frontal bone, heralding the first dissemination of a supraglottic SCC with no extension to other sites. In the most similar case, published by de Bree et al, 2001,^6^ there were no associated symptoms described, and bone involvement occurred in the context of a recurrence, not at the beginning, and without a seizure and an asymptomatic forehead lump at presentation, as in our case (Table 1).

## 5 CONCLUSION

In short, this case illustrates the extremely uncommon presentation of a patient with a painless forehead lump and a seizure owing to extra‐ and intracranial metastases, respectively, as the initial feature of a supraglottic SCC. The conjunction of unexplained significant weight loss, chronic hoarseness or voice change, neurological symptoms (eg, seizure(s)), a forehead lump, and brain metastases should raise suspicion for an advanced supraglottic SCC, in order to ameliorate the disease course.

## CONFLICT OF INTEREST

The authors have no conflict of interest to disclose.

## AUTHOR CONTRIBUTIONS

ML‐R and JB‐L: involved in substantial contributions to conception and design, acquisition of data, or analysis and interpretation of data; drafted the article or revised it critically for important intellectual content; and involved in final approval of the version to be published.
